# Phase I study of samalizumab in chronic lymphocytic leukemia and multiple myeloma: blockade of the immune checkpoint CD200

**DOI:** 10.1186/s40425-019-0710-1

**Published:** 2019-08-23

**Authors:** Daruka Mahadevan, Mark C. Lanasa, Charles Farber, Manjari Pandey, Maria Whelden, Susan J. Faas, Terrie Ulery, Anjli Kukreja, Lan Li, Camille L. Bedrosian, Xiaoping Zhang, Leonard T. Heffner

**Affiliations:** 1 Department of Medicine Division of Hematology/Oncology, University of Arizona Cancer Center, 1515. N. Campbell Avenue, Room 1905, Tucson, AZ 85724 USA; 2 Duke University Medical Center, Durham, NC USA; 3 Summit Medical Center, MD Anderson Cancer Center, Morristown, NJ USA; 4 The West Cancer Center, University of Tennessee, Memphis, TN USA; 5 Alexion Pharmaceuticals, Inc., New Haven, CT USA; 6 Winship Cancer Institute of Emory University, Atlanta, GA USA

**Keywords:** CLL, Multiple myeloma, CD200, Immune checkpoint inhibitor, Samalizumab

## Abstract

**Purpose:**

Samalizumab is a novel recombinant humanized monoclonal antibody that targets CD200, an immunoregulatory cell surface member of the immunoglobulin superfamily that dampens excessive immune responses and maintains self-tolerance. This first-in-human study investigated the therapeutic use of samalizumab as a CD200 immune checkpoint inhibitor in chronic lymphocytic leukemia (CLL) and multiple myeloma (MM).

**Experimental design:**

Twenty-three patients with advanced CLL and 3 patients with MM were enrolled in an open-label phase 1 study (NCT00648739). Patients were assigned sequentially to one of 7 dose level cohorts (50 to 600 mg/m^2^) in a 3 + 3 study design, receiving a single dose of samalizumab intravenously once every 28 days. Primary endpoints were safety, identification of the maximum tolerated dose (MTD), and pharmacokinetics. Secondary endpoints were samalizumab binding to CD200, pharmacodynamic effects on circulating tumor cells and leukocyte subsets, and clinical responses.

**Results:**

Twenty-one patients received > 1 treatment cycle. Adverse events (AEs) were generally mild to moderate in severity. Samalizumab produced dose-dependent decreases in CD200 expression on CLL cells and decreased frequencies of circulating CD200 + CD4+ T cells that were sustained at higher doses. The MTD was not reached. Decreased tumor burden was observed in 14 CLL patients. One CLL patient achieved a durable partial response and 16 patients had stable disease. All MM patients had disease progression.

**Conclusions:**

Samalizumab had a good safety profile and treatment was associated with reduced tumor burden in a majority of patients with advanced CLL. These preliminary positive results support further development of samalizumab as an immune checkpoint inhibitor.

**Trial registration:**

ClinicalTrials.gov, NCT00648739 registered April 1, 2008.

**Electronic supplementary material:**

The online version of this article (10.1186/s40425-019-0710-1) contains supplementary material, which is available to authorized users.

## Introduction

CD200 and CD200 receptor (CD200R) are highly conserved type I paired membrane glycoproteins, consisting of two immunoglobulin (Ig)-like domains (V and C) that belong to the Ig protein superfamily [1–3]. CD200 is widely expressed on a variety of cell types, including B cells, a subset of T cells, dendritic cells, endothelial, neuronal and other cells, while CD200R expression is largely limited to subsets of T cells and myeloid lineage cells [3–7]. The ligation of CD200 with its receptor, CD200R, imparts a multipronged immunosuppressive signal, potently inhibiting T-cell immune responses and natural killer (NK) cytotoxic activity, promoting macrophage secretion of indoleamine-2,3 dioxygenase (IDO), an immunosuppressive tryptophan-catabolizing enzyme, and triggering regulatory T cell (T_reg_) expansion [8–12]. The immune checkpoint function of CD200 on dendritic cells and lymphoid effector cells modulates the activation threshold of inflammatory immune responses and contributes to the maintenance of self-tolerance [13].

CD200 is overexpressed in a wide variety of solid and hematological tumor cell types, including chronic lymphocytic leukemia (CLL) multiple myeloma (MM), acute myeloid leukemia (AML) and others, and is also expressed at elevated levels on cancer stem cells [14–18]. McWhirter et al. first showed that primary tumor cells from CLL patients overexpress CD200 compared with expression on normal B cells [14].

Dampened anti-tumor cytotoxic T cell (CTL) responses are associated with the overexpression of immune checkpoints including CD200, cytotoxic T lymphocyte antigen-4 (CTLA-4) and programmed death-1 (PD-1) on tumor, immune and stromal cells within the tumor microenvironment, and the consequent immunoregulatory signaling events following binding to their respective ligands or receptors [19–21]. Down-regulation of allogeneic Type 1 T helper (Th1) responses, as measured by decreases in interleukin-2 (IL-2) and interferon-gamma (IFN-γ), was noted following the addition of primary CLL cells to an in vitro mixed lymphocyte reaction, and anti-CD200 antibodies reversed this effect, restoring Th1 responses and suppressing T_regs_ [14, 16, 22, 23]. In syngeneic and xenograft murine models, treatment with anti-CD200 antibodies restored lymphocyte mediated anti-tumor responses in vivo [23, 24].

In addition to immunosuppression, overexpression of CD200 on tumor cells has been correlated with aggressive tumor progression, greater metastatic potential, and reduced patient survival, which suggests that CD200 is a promising target for cancer immunotherapy [15, 25]. Accumulated evidence supports the rationale for developing therapeutic anti-CD200 antibodies lacking effector function to block CD200-CD200R-mediated signaling while preserving immune components critical for anti-tumor immunity such as activated T cells and dendritic cells [26]. Blockade of various immune checkpoints, alone or in combination, to reverse T-cell mediated immune suppression and activate anti-tumor immunity is a promising approach to treating cancers [19–21, 27]. Durable clinical responses, including enhanced survival, have been reported with therapeutic blockade of CTLA-4 with ipilimumab, and of PD-1 with pembrolizumab and nivolumab in patients with melanoma, non-small cell lung cancer, renal cancer and head and neck squamous cell carcinoma, leading to FDA approvals [28–35]. Combination therapy blocking both CTLA-4 and PD-1 is now approved for melanoma. Other combinations of targeted therapies, immune checkpoint inhibitors and activators that enhance innate immunity are also being evaluated [36–40].

Samalizumab is a novel recombinant, humanized monoclonal antibody (mAb) that specifically binds to CD200 and blocks its ligation to the CD200 receptor (CD200R). Samalizumab was rationally engineered with an Ig G2/G4 constant region to minimize effector function and preserve immune cell subsets [26].

This is a first-in-human phase I trial to evaluate the safety, pharmacokinetics (PK), pharmacodynamic (PD), and anti-tumor activity of CD200 blockade with samalizumab in patients with CLL and MM, and to identify the maximum tolerated dose (MTD) and dose-limiting toxicity (DLT) of samalizumab.

## Methods

### Eligibility and study schema

This was an open-label, multi-center, sequential cohort dose escalation study (June 2008 - Dec. 2010). The primary endpoints were safety, identification of MTD, and characterization of PK. Secondary endpoints were samalizumab binding to CD200, PD effects on circulating tumor cells and leukocyte subsets, and clinical responses to treatment. The study was conducted in accordance with the Declaration of Helsinki and principles of the International Conference on Harmonisation guidelines on Good Clinical Practice.

Patients with relapsed or refractory CLL or MM, defined as either having failed or refractory to at least one approved therapeutic agent, or who declined standard treatment options, were eligible. Additional inclusion criteria included an Eastern Cooperative Oncology Group performance status score of 0–2 and anticipated survival of > 6 months. Patients were excluded from the study if they met any of the following criteria: absolute neutrophil count < 1000 × 10^9^/L, platelet count < 50,000 × 10^9^/L; pregnant or lactating; prior history of autoimmune hemolysis; immune thrombocytopenia; active autoimmune disease requiring immunosuppressive therapy; positive Coombs’ test; chronic infection with HBV, HCV or HIV; ongoing corticosteroid treatment equivalent to ≥10 mg/day of prednisone; prior stem cell transplantation or prior chemotherapy within 4 weeks or 30 days of enrollment, respectively; neurosurgery or cranial radiotherapy within one year of enrollment; serum creatinine > 1.5 times upper limit of normal, alanine amino transferase or aspartate amino transferase > 2.5 times upper limit of normal, cardiopulmonary disease (New York Heart Association Functional Class III or IV); active systemic bacterial or fungal infection; prior therapy with another investigational product within 30 days of screening; or any condition that could increase the patient’s risk or confound outcome, at the investigators’ discretion.

Patients were assigned sequentially to one of 7 dose level cohorts following a 3 + 3 study design: 50 mg/m^2^, 100 mg/m^2^, 200 mg/m^2^, 300 mg/m^2^, 400 mg/m^2^, 500 mg/m^2^ or 600 mg/m^2^. Each patient only received the dose to which they were assigned. The first dose day was considered as cycle 1, day 0. Patients who tolerated the study drug and had at least stable disease at six weeks following the first dose were permitted to continue therapy until they experienced disease progression, toxicity, or if the investigator or patient wished to discontinue therapy. Additional dosing cycles at the same dose were added as one dose per 28-day cycle, beginning no sooner than six weeks after the initial dose.

At least three patients were assigned per cohort; if none experienced a DLT, escalation to the next dose level occurred with a new cohort. A DLT was defined as any grade 3 or greater toxicity, according to the NCI Common Terminology Criteria for Adverse Events (CTCAE) version 3.0, (NCI 2006) occurring in the first 28 days after dosing in cycle 1. Patients were followed for 10 weeks after their last dose with safety, PK, PD, anti-tumor and clinical response evaluations.

Baseline evaluations for all patients included medical history, physical examination, ophthalmologic slit lamp examination, CBC and differential, chemistry and thyroid panels, electrocardiogram (ECG), hepatitis and HIV serology, Coombs’ test, anti-drug antibody (ADA), coagulation panel, and bone marrow biopsy (optional). CT scans were performed in all CLL patients, while MM patients were evaluated for beta-2 microglobulin, serum protein electrophoresis, serum free light chain and ratio, 24 h urine for total protein and urine protein electrophoresis, serum viscosity, and skeletal survey. See Additional file 1 for further information on dosing and clinical laboratory assays.

### Safety and tolerability

The safety and tolerability of samalizumab in the study patient population were assessed by treatment-emergent adverse events (TEAEs), treatment-emergent serious adverse events (SAEs), clinical laboratory evaluations, vital signs, ECG, and physical and ophthalmology slit lamp examinations.

### Pharmacokinetic assessment

Blood samples for PK analyses in cycle 1 were collected at pre-dose on day 0 (0 h) and at 0.5 h, end of infusion, and 8, 24, 48, 72, 168, 240, 336, 672, and 1008 h after the start of the infusion. Estimated PK parameters for samalizumab, derived from serum concentration-time curves, were total clearance (CL), maximum concentration (C_max_), time to reach C_max_ (T_max_), terminal elimination half-life (T_1/2_), volume of distribution based on terminal elimination phase (V_z_)_,_ and area under the serum concentration-time curve from time zero extrapolated to infinity (AUC_∞_). PK parameters were estimated using non-compartmental methods with WinNonlin® (Version 6.4, Pharsight Corporation, Menlo Park, CA). See Additional file 1 for methodologic details.

### Pharmacodynamic assessment

Blood samples for the measurement of PD markers in cycle 1 were collected pre-dose on day 0, and post-dose on days 1, 7, 14, 24, and 42; during cycles 2 to 4, PD assessments were evaluated pre-dose and on day 14. Samalizumab binding to CD200 on circulating CLL cells was evaluated by multi-parametric flow cytometry using a fluorescently-labeled mAb specific for samalizumab together with a second anti-CD200 mAb specific for an epitope of CD200 distinct from the binding site of samalizumab. CD200 and CD200R expression on peripheral T-cell subsets (CD3+, CD4+, CD8+, activated T cells, T_regs_) collected from CLL and MM patients were evaluated by immunofluorescence and flow cytometry. Data were analyzed as percent of CD200+ cells within the indicated population as well as by mean channel fluorescence intensity (MFI) of bound antibody to reflect the CD200 density on CD200+ cells. See Additional file 1 for methodologic details.

### Cytokine assessment

Serum from patients was evaluated for interleukin (IL)-1β (IL-1β), IL-2, IL-4, IL-6, IL-10, IL-12p70, IFN-γ and tumor necrosis factor alpha (TNF-α) pre-dose and at various times post-dose through week 10 (See Additional file 1 for methodologic details).

### Anti-tumor assessment

Clinical responses were based on the Modified NCI Working Group Response Criteria for CLL [41] and on the International Myeloma Working Group Uniform Response Criteria for MM [42]. For CLL, the overall response rate (ORR) was defined as the percentage of patients who maintained their best response for at least one month after achieving that best response and having either a complete response (CR), partial response (PR), nodular partial response (nPR), or stable disease (SD). Progressive disease (PD) was defined by one of the following: > 50% increase in the sum of the products of at least two lymph nodes (at least one lymph node must be > 2 cm), appearance of new lymph nodes, > 50% increase in the size of the liver and/or spleen, > 50% increase in the absolute number of circulating lymphocytes to at least 5000/uL, or transformation to a more aggressive histology (Richter’s Syndrome). For MM, ORR was defined as the percentage of patients who had sCR (stringent CR), CR, very good partial response (VGPR), or PR on two consecutive assessments made at any time before the administration of any new therapy. PD was defined as > 25% increase of urine M-protein.

Computed tomography (CT) scans of the neck, chest, abdomen, and pelvis in CLL patients were evaluated using sum of the products of bi-dimensional measurements of all target lesions [41], Additional cycles of treatment were continued if there was evidence of response by blood counts or physical exam at weeks 4 and 8. Anti-tumor responses were evaluated as the percent change from baseline in lymphadenopathy.

### Statistical analyses

Patients who received at least one dose of samalizumab were included in safety, PK, PD, and clinical response- analyses. Data collected at all sites were pooled for analysis, and descriptive statistics were used to summarize the data. All tables and listings were generated using SAS® Version 9.2 or higher (SAS Institute, Inc., Cary, NC).

## Results

### Patient disposition and treatment exposure

Twenty-six patients, 23 with CLL (4 were treatment naïve) and 3 with MM, were enrolled from June 2008 to December 2010 across four study sites. Patient characteristics are given in Table 1. All 26 patients received at least one samalizumab dose. The clinical study was amended to allow multiple doses of samalizumab to be administered. Twenty-one patients (81%) received multiple dosing cycles and five patients (19%), including two MM patients, received one dose. Thirteen patients (50%) received ≥4 cycles of samalizumab. The maximum number of cycles received by any patient was 18 (300 mg/m^2^ dose cohort). The study was terminated prematurely by the sponsor for administrative reasons. Data from all 26 patients were analyzed except where noted.

**Table 1:** Patient characteristics

Parameter	50 mg/m^2^ (*N* = 4)	100 mg/m^2^ (*N* = 5)	200 mg/m^2^ (*N* = 3)	300 mg/m^2^ (*N* = 3)	400 mg/m^2^ (*N* = 3)	500 mg/m^2^ (*N* = 7)	600 mg/m^2^ (*N* = 1)	Total (*N* = 26)	%
Gender									
Male	3	3	2	1	3	6	0	18	69
Female	1	2	1	2	0	1	1	8	31
Race									
Caucasian	3	4	3	3	2	6	1	23	89
Black	0	1	0	0	1	1	0	3	11
Age (years, at screening)									
Mean (SD)	64.645 (11.7737)	60.716 (13.5373)	62.387 (20.6538)	68.227 (15.4956)	74.25 (11.0807)	67.104 (9.5240)	65.92 (NA)	65.9 (12.14)	
Median	66.92	59.43	61.370	70.500	69.00	66.98	65.92	66.9	
Range	49.84–74.9	41.0–77.3	42.26–83.53	51.72–82.76	66.77–86.98	53.65–79.69	65.92	41–87	
Type of malignancy									
CLL	4	4	4	3	3	5	0	23	89
Multiple Myeloma	0	0	0	0	0	2	1	3	11
Time from diagnosis to first samalizumab dose (days)									
Mean (SD)	3672.3 (3323.28)	1556.6 (1571.86)	3017.0 (912.22)	1577.3 (1010.13)	3371.0 (2045.69)	1850.4 (1190.99)	1451.0 (NA)	233 (1817.1)	
Median	2510.5	813.0	2922.0	1264.0	3061.0	1649.0	1451.0	1887	
Range	1148–8520	43–4200	2156–3973	761–2707	1498–5554	154–3123	1451–1451	154–8520	
Patients with previous chemotherapy	3	3	3	3	2	7	1	22	85
Patients with previous radiation	0	0	0	0	0	1	1	2	8
Patients without prior chemotherapy or radiation treatments	1	2	0	0	1	0	0	4	15
Study completion									
Yes	2	3	0	1	1	2	0	9	34.6
No	2	2	3	2	2	5	1	17	65.4
Reason for non-completion									
Treatment-emergent adverse event	1	0	1	2	1	0	0	5	19.2
Patient requested to withdraw	1	0	1	0	0	2	0	4	15.4
Lack of efficacy	0	0	0	0	1	2	0	3	11.5
Investigator considered it advisable/in the patient’s best interest	0	0	0	0	0	1	1	2	7.7
Patient did not complete follow up	0	1	0	0	0	0	0	1	3.8
Death (not related to study drug)	0	1	0	0	0	0	0	1	3.8
Positive antidrug antibody serology	0	0	1	0	0	0	0	1	3.8

### Safety and adverse events

The MTD was not reached, and administration of samalizumab from 50 to 600 mg/m^2^ was well-tolerated in patients with CLL or MM. Only one patient was treated with the 600 mg/m^2^ dose; this patient (with MM) did not complete the study and died of progressive disease shortly after two weeks of follow-up. A total of 256 TEAEs were reported by 25 (96%) patients; the most commonly reported TEAEs are listed in Table 2. Five patients experienced TEAEs that were deemed possibly, probably, or definitely related to study drug that were grade 3–4 in severity. The most common drug-related grade 3–4 TEAEs were blood and lymphatic system disorders (anemia, neutropenia, and thrombocytopenia) reported in three patients (12%). The other drug-related grade 3–4 TEAEs were reduced visual acuity and muscular weakness (both in the same patient, 4%), respiratory syncytial virus infection (1 patient, 4%), and rash (1 patient, 4%) (Table S1). TEAEs that were considered definitely related to the study drug occurred in two of three patients with elevated ADA at the time of samalizumab administration: hypersensitivity (grade 1 allergic reaction) and urticaria (grade 2 hives).

**Table 2:** Treatment-emergent adverse events (TEAEs) reported in ≥5% patients by organ system

System Organ Class	Samalizumab Treatment Group							
	50 mg/m^2^ *N* = 4	100 mg/m^2^ *N* = 5	200 mg/m^2^ *N* = 3	300 mg/m *N* = 3	400 mg/m^2^ *N* = 3	500 mg/m^2^ *N* = 7	600 mg/m^2^ *N* = 1	Overall *N* = 26
General Disorders and Administration Sites								11 (42)
Fatigue	1 (25)	–	1 (33)	3 (100)	–	1 (14)	–	
Peripheral coldness	1 (25)	–	–	–	–	–	–	
Pyrexia	–	–	–	–	–	1 (14)	–	
Chills	–	–	–	–	–	1 (14)	–	
Edema	–	–	–	2 (67)	–	–	–	
Skin and Subcutaneous Tissue								9 (35)
Erythema	–	–	–	1 (33)	–	–	–	
Night sweats	–	–	–	–	–	1 (14)	–	
Pruritus	1 (25)	1 (20)	–	–	–	–	–	
Rash	1 (25)	1 (20)	1 (33)	1 (33)	–	–	–	
Urticaria	1 (25)	–	–	–	–	–	–	
Gastrointestinal								5 (19)
Abdominal distension	–	–	–	1 (33)	–	–	–	
Abdominal Pain	–	–	–	2 (67)	–	–	–	
Diarrhea	1 (25)	1 (20)	–	–	–	–	–	
Infections and Infestations								4 (15)
Upper Respiratory Tract Infection	–	1 (20)	–	–	1 (33)	1 (14)	–	
Abscess	–	–	–	1 (33)	–	–	–	
Musculoskeletal and Connective Tissue								4 (15)
Arthralgia	–	–	–	1 (33)	–	–	–	
Muscular weakness	1 (25)	–	–	–	–	–	–	
Myalgia	1 (25)	–	–	–	–	–	–	
Stiffness	–	1 (20)	–	–	–	–	–	
Nervous System								3 (12)
Dizziness	–	–	–	1 (33)	–	–	–	
Headache	–	–	–	–	–	1 (14)	–	
Paraesthesia	–	–	–	1 (33)	–	–	–	
Blood and Lymphatic System								7 (27)
Anemia	–	–	–	1 (33)	1 (33)	–	–	
Neutropenia	2 (50)	–	–	1 (33)	1 (33)	–	–	
Thrombocytopenia	–	–	–	–	1 (33)	–	–	
Eye								6 (23)
Eye pain	1 (25)	–	–	–	–	1 (14)	–	
Night blindness	1 (25)	–	–	–	–	–	–	
Photophobia	1 (25)	–	–	–	–	1 (14)	–	
Reduced visual acuity	1 (25)	–	–	–	–	–	–	
Laboratory	–		–		–	–	–	2 (8)
Increased blood viscosity		–		1 (33)		–		
Decreased platelets		1 (20)				–		
Respiratory, Thoracic and Mediastinal								4 (15)
Cough	–	–	–	–	–	1 (14)	–	
Dyspnea	–	–	–	1 (33)	–	1 (14)	–	
Pulmonary edema	–	–	–	–	–	1 (14)	–	

Values in parentheses are the percentage of patients

Only one occurrence per patient counted for each category

“-” indicates zero

Of the 26 study participants, six (23%) experienced at least one SAE; four (15%) had SAEs considered unrelated to study drug and two (8%) had SAEs considered possibly related to study drug. One fatal SAE, due to complications post-elective cholecystectomy and ensuing renal failure, occurred 23 days after the fourth dose of 100 mg/m^2^. The investigators determined that the event was unrelated to samalizumab. No SAEs led to discontinuation.

In some patients, ECGs revealed heart rate, PR interval, QRS duration and QTc intervals outside normal ranges on occasion, but these were not clinically significant events. In aggregate, no QT interval changes were observed. No significant ophthalmologic findings were attributed to samalizumab treatment.

### Pharmacokinetics

Following a single intravenous dose of samalizumab (100–600 mg/m^2^), the mean T_max_ values across all dose levels ranged from 1.23 to 8.93 h, the mean T_1/2_ for samalizumab increased from 85.1 h to 537.9 h (3.5 to 22.4 days), and mean systemic CL showed a decreasing trend in the three highest dose cohorts (Table 3). The mean V_z_ did not appear to be dose related. C_max_ increased in a dose-proportional manner and AUC_∞_ increased in a more than dose-proportional manner. For C_max,_ the β value was 1.01 (95% CI: 0.85–1.17) and for AUC_∞_, the β value was 2.01 (95% CI: 1.59–2.42). The serum concentration-time profiles of samalizumab are graphed as the mean serum concentration of samalizumab after the first intravenous administration at the indicated doses. Error bars represent the standard deviation (Additional file 1: Figure S1).

**Table 3:** Summary of samalizumab PK parameters

Dose		No. of patients	T_max_ (h)	C_max_ (μg/mL)	AUC_∞_ (μg·h/mL)	T_1/2_ (h)	CL (mL/h)	V_z_ (mL)
100 mg/m^2^		5	2.21 ± 3.25	38.9 ± 4.75	2792 ± 2227	85.1 ± 60.9	101 ± 67.5	8246 ± 1499
200 mg/m^2^		3	1.23 ± 0.09	90.2 ± 10.6	11,957 ± 6599	107 ± 45.2	36.3 ± 14.9	4943 ± 469
300 mg/m^2^		3	4.71 ± 4.65	109 ± 42.7	36,636 ± 11,540	371 ± 48.9	16.9 ± 4.15	9186 ± 3215
400 mg/m^2^		3	8.93 ± 11.1	135 ± 37.8	37,679 ± 7219	245 ± 33.9	21.3 ± 4.61	7391 ± 897
500 mg/m^2^		7	3.87 ± 1.85	211 ± 44.9	62,898 ± 24,222	365 ± 172	19.5 ± 10.4	8490 ± 1715
600 mg/m^2^		1	3.08	288	134,629	538	7.58	5880

Values are presented as Mean ± SD. The samalizumab serum concentration assay had a lower limit of quantification of 3.70 μg/mL and the standard curve ranged from 3.7 to 100 μg/mL Assay precision was 1 to 18% and accuracy was 93.2 to 127.8% (Mean % of recovery)

### Pharmacodynamics

The binding of samalizumab to CD200 on peripheral CLL cells was evaluated in 21 of 23 (91%) CLL patients. Two patients were not evaluable because of insufficient circulating CLL cells and high background level staining precluding reliable analysis. Despite considerable inter-patient variability in baseline peripheral CLL counts (range 0.8–90.7%), nearly all CLL cells (85 to 100%) were CD200+, although there was wide interpatient variation in the intensity of CD200 expression on CLL cells.

**Fig. 1:**
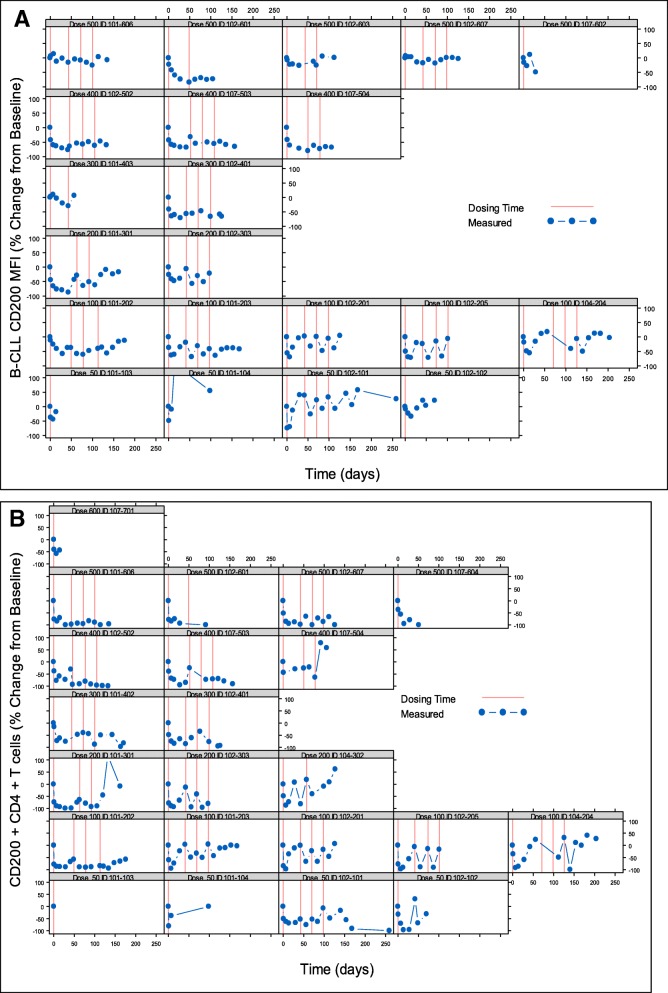
Each panel displays data for a single patient (indicated at the top of each graph) at baseline (Day 0) and after samalizumab dosing at the indicated time points. For simplicity, no more than the first 4 dosing cycles are shown. **a**. Percent change from baseline in CLL CD200 expression (mean channel fluorescence (MFI)) in CLL patients. **b**. Percent change from baseline in CD200+ CD4+ T cells (%) in CLL and MM patients

On day 1 after dosing, bound samalizumab was detected on peripheral CD200+ CLL cells in 16 of 21 (76%) evaluable patients. Increased binding was observed at higher doses (200–500 mg/m^2^). The range of frequencies of CLL cells with bound samalizumab on day 1, and the density of bound samalizumab MFI by dose cohort are summarized in Table 4. Down-regulation of CD200 expression on CLL cells was observed in 18 of 21 patients (86%) after samalizumab dosing (Fig. 1a). The density of CD200 expression (MFI) on day 1 was reduced from baseline by 6.8–74.3%. A dose-dependent reduction in CD200 expression on CLL cells was observed after multiple dosing: transient reductions in CD200 expression were generally observed in patients treated with lower doses (50–200 mg/m^2^), whereas sustained reductions were seen in 18 of 21 evaluable patients (86%) patients receiving higher doses (300–500 mg/m^2^).

**Table 4:** Samalizumab bound to CD200+ CLL cells by cohort

Samalizumab Cohort	CLL cells bound by samalizumab (%)		Density of bound samalizumab (MFI)	
	Pre-dose	Day 1	Pre-dose	Day 1
50 mg/m^2^ (*n* = 4)	0.3–0.7	1.1–3.3	1.9–3.1	3.3–4.1
100 mg/m^2^ (*n* = 5)	0.2–2.4	0.2–9.5	3.5–5.6	5.0–11.1
200 mg/m^2^ (*n* = 2)	0.3–0.7	27.8–29.6	3.2–3.3	16.6–19.3
300 mg/m^2^ (*n* = 2)	0.5–0.7	5–28.6	1.7–3.0	5.6–16.8
400 mg/m^2^ (*n* = 3)	1.1–5.7	1.7–71.3	1.7–3.4	4.6–26.6
500 mg/m^2^ (*n* = 5)	0.5–2.1	1–47.0	1–3.7	1.9–17.6

Binding of samalizumab to CD200 on circulating CLL cells was evaluated by multi-parametric flow cytometry using a fluorescently-labeled monoclonal antibody specific for samalizumab (7B8) together with a second anti-CD200 antibody (IB2) specific for an epitope of CD200 distinct from the binding site of samalizumab

The percent change from baseline in peripheral CD200+ CD4+ T cells for all evaluable CLL and MM patients is shown in Fig. 1b. Of the 26 enrolled patients, 21 (81%) were evaluated; four patients with CLL and one with MM were not evaluable due to insufficient circulating immune cells. By day 1 after samalizumab dosing, all evaluable patients showed a decrease in the frequency of peripheral CD200+ CD4+ T cells (range of − 15.6% to − 85.3% from baseline). Of 17 patients who received > 1 dose of samalizumab, 16 (94%) continued to show reductions in CD200+ CD4+ T cell frequencies in response to dosing. Similar to the reduction in CD200 expression observed on CLL cells, a dose-dependent reduction in the frequencies of peripheral CD200+ CD4+ T cells was also observed, with transient responses at low doses (50–200 mg/m^2^) and sustained responses at higher doses (300–500 mg/m^2^).

With the exception of CD200+ CD4+ T cells, no apparent dose-dependent effect of samalizumab on other T-cell subsets was found. Changes in the frequencies of CD3+ cells or total CD4+ cells (regardless of CD200 expression) revealed considerable inter-patient variability across and within cohorts, with no clear trends discernable. Cell counts of CD8 + cells, activated T cells, and T_regs_, at baseline or during treatment, were too low to provide reliable results. A notable exception is Patient #102–502, treated at the 400 mg/m^2^ dose, who had sufficient immune cells for analysis; this patient is discussed in the Additional file 1 (pages 8–10). Patients with MM received up to three doses of samalizumab and showed little change in T-cell subsets.

In one patient, a transient increase in peripheral B-CLL count, absolute lymphocyte count and white cell count was observed following initial samalizumab treatment (Additional file 1: Figure S2). The observed binding to CD200+ B-CLL cells and the reduction in CD200 expression indicates that samalizumab is binding to and blocking its intended target, the immunoregulatory molecule CD200. However, even at doses of 500 mg/m^2^, neither maximal saturation of CD200 binding nor maximal sustained decreases in CD200 expression on the B-CLL target cells was achieved. Changes from Baseline in absolute lymphocyte count and circulating B-CLL cells were found to trend similarly: an overall reduction in peripheral B-CLL cells after samalizumab dosing paralleled the reduction in absolute lymphocyte count. In 14/23 (56.5%) patients, this increase was followed by a reduction in both peripheral CLL cells and absolute lymphocyte count with multiple samalizumab doses (% decrease 0.5 to 50%).

Detectable levels of Th1 and Th2 cytokines (IFN-γ, IL-2, IL-10, IL-12p70 and TNF-α) were observed following the first dose, but levels were neither sustained nor associated with clinical symptoms (data not shown).

### Response to therapy

The ORR for CLL patients was 4% (1 of 23) with this conservative dosing schedule. Sixteen CLL patients (70%) achieved SD, and five patients (22%) had PD. One patient was not evaluable and one patient had a PR that was confirmed at cycle 12 (Patient #102–502; see case study in Additional file 1 – pages 3–5 and 8–10). Patient #102–502 was a newly diagnosed with Rai stage IV and was treated at the 400 mg/m^2^ dose. A reduction in CD200 expression on CLL cells was associated with a transient increase in peripheral CLL cells followed by a progressive reduction peripheral CLL cells (Fig. S2), CD200+ CD4+ T cells and T_REG_s. In contrast, CD8+ T-cells increased indicating an anti-tumor immune response (Additional file 1: Figure S3). Reduced CD200 expression on CLL cells paralleled a reduction in bulky lymphadenopathy (Additional file 1: Figure S4). One patient, maintained SD through cycle 18 (300 mg/m^2^) and two patients maintained a SD through cycle 6 (500 mg/m^2^) when the study was terminated. All 3 MM patients had PD.

**Fig. 2:**
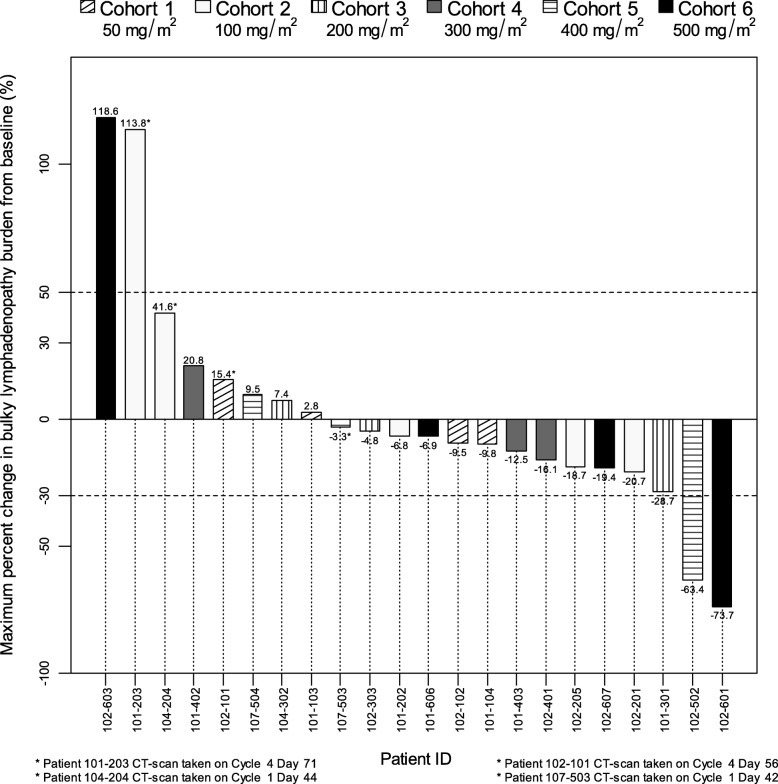
Each vertical bar represents the maximum change obtained for a single patient, identified by the six digit code at the bottom of the graph, that had a baseline CT scan and at least one subsequent scan. The horizontal dotted line at 50% represents a cut-off above which lymph node enlargement represents progressive disease whereas the horizontal dotted line at − 30% represent a cut-off below which lymph node regression represents clinically significant improvement. Patient 107–602 (500 mg/m^2^ cohort) did not have a post-dose CT scan and was not evaluable

Of the 22 patients whose primary target lesions were measured by CT scans at baseline and at least one subsequent scan after dosing with samalizumab, 14 (64%) had a decrease in tumor burden post-dosing. Twelve of these patients were from all dose level cohorts and had a maximum decrease in lymphadenopathy ranging from 3.3 to 28.7%. Two patients had a > 50% reduction in the total amount of lymphadenopathy: these patients were from the two highest dose cohorts (400 and 500 mg/m^2^) and had maximum decreases in lymphadenopathy of 63.4 and 73.7%, respectively. A 30% decrease in total lymphadenopathy was the cut-off below which lymph node regression was considered a clinically significant improvement. The maximum change in lymphadenopathy in individual patients is shown in Fig. 2.

Eight patients from all dose level cohorts had a maximum increase in lymphadenopathy (range: 2.8 to 118%). Two of these patients had a > 50% maximum increase in lymphadenopathy, a cut-off above which lymph node enlargement represents progressive disease.

Twenty of the 22 patients (91%) showed a decrease in the size of at least a single lesion. Ten (45%) had a maximum reduction > 40% and five (23%) had > 50% reduction. The reductions in individual lesions did not always correlate with a similar reduction in the sum of the bi-dimensional products of target lesions at the same time point. Eight patients (36%) had an increase in the sum of products of bi-dimensional target lesions at the time they experienced a maximum reduction in a single lesion (Table 5).

**Table 5:** Maximum reduction in the sum of lesions and a single lesion after samalizumab dosing

Samalizumab Treatment Group	Patient ID	Single Lesion^b^		Sum of Lesions^b^	
		% Change	Cycle Day	% Change	Cycle Day
50 mg/m^2^ (*N* = 4)	101–103	−3.45	C1 D28	2.77	C1 D28
	101–104	−45.29	C1 D77	−9.8	C1 D35
	102–101	−19.75	C4 D16	4.32	C4 D16
	102–102	−9.77	C1 D35	−9.46	C1 D35
100 mg/m^2^ (*N* = 4)^a^	101–202	−37.78	C4 D0	−6.83	C4 D0
	101–203	−11.54	C4 D0	7.0	C1 D28
	102–201	−95.31	C4 D0	−20.7	C4 D0
	102–205	−41.36	C3 D26	−18.7	C3 D26
200 mg/m^2^ (*N* = 2)^a^	101–301	− 48.67	C1 D28	−28.7	C1 D28
	102–303	−42.05	C3 D21	−4.75	C3 D21
300 mg/m^2^ (*N* = 3)	101–402	−14.29	C4 D43	8.3	C4 D43
	101–403	−23.81	C1 D29	−12.5	C1 D29
	102–401	−58.73	C1 D35	−16.1	C16 D1
400 mg/m^2^ (*N* = 3)	102–502	−87.02	C13 D35	−63.4	C13 D35
	107–503	−54.55	C4 D27	−3.34	C1 D42
	107–504	−29.34	C1 D29	9.52	C1 D29
500 mg/m^2^ (*N* = 4)^a^	101–606	−48.15	C6 D0	−6.91	C6 D0
	102–601	−86.36	C2 D28	−73.7	C2 D28
	102–603	−5.13	C1 D25	1.84	C1 D25
	102–607	−37.36	C1 D28	−19.4	C1 D28

^a^Patient 107–602 (500 mg/m^2^ cohort) did not have a post-dose CT scan and was not evaluable; Patients 104–204 (100 mg/m^2^ cohort) and 104–302 (200 mg/m^2^ cohort) had no reduction in any of their target lesions and are not included

^b^Maximum reduction for a single lesion (product of bi-dimensional tumor measurement) and maximum reduction for sum of lesions (sum of the products of bi-dimensional tumor measurements) are presented as a % Change from baseline on the indicated Cycle and Day

## Discussion

Although promising novel therapies have recently become available, the majority of patients with CLL and MM will ultimately relapse or become refractory to currently available therapeutic regimens, and the only known curative therapy for CLL and MM is stem cell transplantation, with its associated high morbidity and mortality [43, 44]. Blockade of the CD200-CD200R immune checkpoint using a therapeutic anti-CD200 mAb was hypothesized to restore and/or enhance tumor cell recognition and CTL mediated anti-tumor responses in advanced CLL and MM patients with limited therapeutic options. Samalizumab is a novel, first-in-class, recombinant humanized anti-CD200 mAb, engineered to ablate effector function, that specifically binds to the immune checkpoint CD200, blocks receptor engagement and signaling and antagonizes CD200-driven immune suppression, thereby allowing the patient’s immune surveillance to detect tumor cells and mount an anti-tumor immune response.

Samalizumab at doses of 50 to 600 mg/m^2^ was well tolerated, MTD was not determined and no DLTs were observed. TEAEs were generally mild or moderate in severity and, overall, were considered manageable. None of the patients discontinued samalizumab treatment due to SAEs and the frequency of SAEs appears not to be dose-related. However, two patients discontinued participation in the post-dose follow-up period due to SAEs that were unrelated to study drug. Samalizumab dosing was not associated with clinically significant cytokine responses at any time (data not shown). ADA frequency was consistent with that in literature reports of other therapeutic mAbs [45, 46].

The mean T_1/2_ for samalizumab increased with increasing doses (100 mg/m^2^–600 mg/m^2^). The high clearance of samalizumab at low doses likely represents saturation binding to membrane-associated CD200 surface antigen. At higher doses, antibody clearance is likely due to nonspecific elimination through the cells of the reticuloendothelial system [47]. These findings are consistent with those of other therapeutic mAbs reported in the literature [48].

Samalizumab binding dampens CD200 overexpression on CLL cells in a dose-dependent manner, and the reduction in CD200 expression is sustained at higher doses (300–500 mg/m^2^). However, it is apparent from these early data that the concentrations of samalizumab achieved in this phase I study were insufficient to completely saturate cell-surface CD200 on the CLL cells. The PK and PD assays incorporated in this study may provide a strategy to guide optimal dosing in future trials. Sustained decreases in CD200 expression on peripheral CD200+ CD4+ T cells (reduction in the percentage of CD200+ CD4+ T cells) is observed in CLL and MM patients at higher doses (300–600 mg/m^2^). No other dose-dependent changes in T-cell subsets were consistently observed, although one patient with no prior chemotherapy demonstrated the predicted immunomodulatory changes following multiple doses of samalizumab: increases in frequencies of activated T cells and CD8+ T cells during prolonged samalizumab treatment, with concomitant reduction of T_REGs_ (Additional file 1 pages 3–5 and 8–10).

The clinical responses reported support the study’s central hypothesis that blockade of the immune inhibitory ligand CD200 by samalizumab promotes anti-tumor activity: serial CT scans revealed that more than half (64%) of evaluable CLL patients had reductions in tumor burden, with two patients having > 50% reduction. Most CLL patients had a decrease in size of at least one lesion, with 22.7% of CLL patients experiencing a reduction of > 50% in at least one lesion. However, in 8 patients, 4 of whom had SD, the maximum reductions in a single lesion did not always correlate with the maximum reduction in the sum of the products of all lesions at the same time point. This may represent an immune-modulated response consistent with pseudoprogression [49]. In clinical trials of solid tumors, increases in tumor burden that can precede responses led to novel evaluation criteria (immune-related response criteria (irRC)) [49]. An increase in tumor burden prior to response evaluation may reflect either continued tumor growth until a sufficient immune response develops or transient immune-cell infiltrate. Similar patterns of stable disease or improvement after an initial increase in tumor burden have been observed with other immune checkpoint inhibitors such as ipilimumab and anti-PD-1 mAbs [28, 32, 33]. Although the irRC have been implemented in solid tumors, mechanisms underlying these increases may also apply to lymphadenopathy in B-cell malignancies.

SD was achieved in sixteen CLL patients: one patient received 18 cycles of samalizumab and maintained SD through cycle 18 (300 mg/m^2^) and two patients maintained SD through cycle 6 (500 mg/m^2^). All 3 patients remained on samalizumab until the trial was concluded. One treatment-naïve Rai Stage IV CLL patient (Patient# 102–502) who received 13 cycles of samalizumab achieved a durable PR lasting for > 6 years with no further interventions and is reported to be healthy at the time of this manuscript. This patient may have had a superior clinical response to samalizumab because of his preserved immune function prior to treatment.

## Conclusions

Advances in the understanding of the mechanisms of protective anti-tumor immunity has led to the development of immune checkpoint therapy with mAbs targeting inhibitory pathways that normally suppress anti-tumor T-cell immunity and mediate immune tolerance. The findings of this study provide proof-of-concept for targeted inhibition of the immune checkpoint CD200, as samalizumab appears to have provided significant therapeutic benefit to some CLL patients despite a sub-optimal dosing schedule. These findings support clinical investigation of samalizumab in CLL and other tumor types with elevated CD200 expression. Further clinical investigation should include additional dosing regimens, including further dose-escalation and more frequent dosing of samalizumab and/or potential combinations with other FDA-approved targeted or immunomodulatory agents.

## Additional file

Additional file 1: Supplemental data on the Phase I Samalizumab trial of CLL and MM. (DOCX 799 kb)

## Data Availability

Supporting data is available at the journals request.
