# Supplementary material for: Phase I study of samalizumab in chronic lymphocytic leukemia and multiple myeloma: blockade of the immune checkpoint CD200
Source: J Immunother Cancer. 2019 Aug 23;7:227. doi: 10.1186/s40425-019-0710-1 (PMC6708181; doi:10.1186/s40425-019-0710-1)

**Additional file**

**Detailed Methods**

**Eligibility and study schema**

Samalizumab was diluted with an equal volume of 0.45% Sodium Chloride Injection, US Pharmacopeia, and infused intravenously at a rate of 125 mL/h. All doses of samalizumab were individualized to the patient’s body surface area in milligrams per square meter (mg/m^2^), based on height and weight at screening.

If any of the initial 3 patients in a cohort experienced a dose limiting toxicity (DLT), the cohort was to be expanded to at least 6 patients. If less than one-third of patients within an expanded cohort experienced a DLT, escalation to the next dose level was to occur with a new cohort. If at least one-third of patients within the expanded cohort experienced a DLT, then no further dose escalation was to occur. However, if the DLTs were inconsistent with those seen in other patients receiving samalizumab, additional patients (up to a maximum of 12) could be enrolled in the cohort. Dose escalation did not occur in the next cohort until 1) after the initial three patients from the previous dose cohort had completed the week 4 study visit; 2) all grade 3 and/or 4 toxicities, if any, had resolved; 3) safety parameters had been reviewed; and 4) no maximum tolerated dose (MTD) had been established in the previous cohorts.

If additional doses of samalizumab were administered, all baseline evaluations were obtained on the day of dosing and the final follow-up visit, except for the ophthalmologic slit lamp examination (only at the final follow-up visit). All clinical laboratory assessments related to safety and efficacy, including the cytokine assay, were performed by Esoterix Clinical Trial Services (Cranford, NJ), except the ADA, which was performed by Alexion Pharmaceuticals, Inc., (Cheshire, CT).

**Pharmacokinetic assessment**

The following PK parameters were estimated using noncompartmental analysis method after a single IV dose of samalizumab: clearance (CL), maximum observed serum concentration at the end of infusion (Cmax), time to reach Cmax after drug administration (Tmax), elimination half-life (T1/2), volume of distribution at the terminal elimination phase (Vz), area under the serum concentration-time curve from time zero to the last detectable time point (AUClast), and area under the serum concentration-time curve from time zero to infinity (AUCinf). (Note that AUClast was calculated using the linear trapezoidal rule, and AUCinf was estimated from log-linear regression of the elimination phase data points). The following PK parameters were estimated after multiple-dose administration given at a minimum of 28-day cycles: Cmax, observed serum concentration on Day 14 after dosing (C14), and accumulation ratio after multiple dosing for Cmax and C14 (Rac). Samples were analyzed for serum samalizumab concentrations at Alexion Pharmaceuticals, Inc. using a validated Enzyme Linked Immunosorbent Assay (ELISA). The range of the analytical assay used to determine serum samalizumab concentrations was 0.137-100 μg/mL (See Figure S1).

A power analysis was conducted to assess dose proportionality of samalizumab following first dose administration. (Gough K, Hutchison M, Keene O, et al. Assessment of dose proportionality: report from the statisticians in the pharmaceutical industry/pharmacokinetics UK Joint working party. *Drug Info J*. 1995; 29:1039-1048.) In the power analysis used to assess dose proportionality of samalizumab, the natural logarithm (Ln) C_max_ and AUC_∞_ values were regressed against Ln dose values. The 95% confidence interval (CI) was constructed for β value and inclusion of null value of 1.0 within the 95% CI was considered to suggest that a dose-proportional increase could not be ruled out.

**Pharmacodynamic assessment**

Binding of samalizumab to CD200 on circulating CLL cells was evaluated by multi-parametric flow cytometry using a fluorescently-labeled antibody specific for samalizumab (7B8; Alexion Pharmaceuticals, Inc.) together with a second anti-CD200 antibody specific for an epitope of CD200 distinct from the binding site of samalizumab (1B2; Alexion Pharmaceuticals, Inc.). Binding studies were performed by Esoterix Clinical Trail Services. Changes in CD200 expression were detected by evaluating binding of IB2 to CLL cells or CD4+ T cells by multiparametric flow cytometry and immunofluorescence.

**Anti-drug antibody (ADA) assessment**

The potential development of antibodies directed against samalizumab was evaluated in serum using a validated electro-chemiluminescence assay (Alexion Pharmaceuticals, Inc.) Samples having a positive signal in a screening assay were reevaluated in a confirmatory assay in which the specificity of ADA was verified by the addition of excess unlabeled samalizumab.

**Cytokine assessment**

Circulating cytokines were measured in serum using a cytometric bead array assay (Esoterix Clinical Trial Services). Serum samples were taken pre-dose on day 0, and post-dose on days 1-3, and weeks 1, 2, 4, 6, 8, and 10, and at other times as clinically indicated and at the end of the study. Samples were analyzed for IL1-β, IL-2, IL-4, IL-6, IL-10, IL-12p70, IFN-γ and TNF-α.

Results

Case study of Patient # 102-502

Patient # 102-502 is a Caucasian man (94.1 kg, 182.9 cm), aged 66 at study entry (2009) and diagnosed with CLL in July 1994. He presented with worsening fatigue, splenomegaly, adenopathy, and lymphocytosis. Past medical history included allergies (animal and seasonal), allergic rhinitis, pruritic and flaking scalp, and dermal cysts on his back. The major laboratory finding at the time of diagnosis was significant thrombocytopenia. After diagnosis, the patient experienced grade 2 neutropenia, herpes zoster, gastroesophageal reflux disorder, nocturia and fatigue, as well as a small umbilical hernia and mild diverticulosis. All of these conditions were ongoing at the time of enrollment. In 2006, the patient was diagnosed with early stage colon and prostate cancer that were successfully treated surgically. The patient had received no prior therapy for CLL.

At study entry in 2009, the patient had advanced CLL disease (Rai stage 4) as evidenced by splenomegaly, thrombocytopenia and lymphadenopathy confirmed by CT scan. Laboratory evaluations showed a platelet count of 85 x 10^9^/L (range 140-415 x 10^9^/L), an absolute lymphocyte count of 18.84 x 10^9^/L (range 0.7-4.5 x 10^9^/L), and hemoglobin at 12.8 g/dL (range 11.5-15 g/dL). The patient had a new laboratory finding of hyperuricemia. Other routine laboratory parameters were normal. A bone marrow biopsy revealed ~90% of total cells consisting of CLL and absent iron stores. At baseline, approximately 73.6% of leukocytes were identified as CLL cells by flow cytometry.

The baseline CT scan revealed multiple adenopathies, 6 of which were selected as target lesions for follow-up on serial images. At screening, 2 lesions were identified in the left axillary lymph node chain (5.78 cm^2^ and 5.72 cm^2^), and single lesions were seen in the aortacaval lymph node (2.42 cm^2^), right axillary lymph node (3.22 cm^2^), subcarinal lymph node (3.64 cm^2^), and right inguinal lymph node (2.24 cm^2^).

The patient was assigned to the 400 mg/m^2^ cohort and received the first dose of samalizumab on September 28, 2009 and the last dose on September 20, 2010. He received a total of 13 cycles of treatment. He received a dose of 866 mg each cycle and tolerated the treatment well, with no adverse events.

After the first samalizumab dose, CD200 expression on peripheral CLL cells was transiently reduced, but on subsequent dosing a sustained reduction was observed (see Patient 102-502 panel in Figure1A). Coincident with the reduction in CD200 expression on CLL cells, the patient showed an initial, transient increase in peripheral CLL cells followed by a progressive reduction of both peripheral CLL cells (Figure S2) and CD200+ CD4+ T cells (see Patient 102-502 panel in Figure 1B). Reduced CD200 expression on CLL cells paralleled a reduction in bulky lymphadenopathy (Figure S4 and described below). Transient increases in absolute lymphocyte count and white blood cell count were also observed. CT scan findings showed substantial decrease from baseline (32%) in bulky lymphadenopathy on day 28 (cycle 1 day 28). Another CT scan on day 28 revealed that the size of the largest target lesion in the left axillary lymph node was reduced by 58.48%, (5.78 cm^2^ vs. 2.40 cm^2^, baseline vs. day 28, respectively) (Figure S4). At this time, the platelet count was 124 x 10^9^/L (range 140-415 x 10^9^/L), and had improved 26.5% from pre‑dose value of 98 x 10^9^/L, but continued to have a marked leukocytosis with a white blood cell count of 48.80 x 10^9^/L. Hemoglobin and other routine laboratory values were within the normal range.

Transient declines in the frequencies of CD4+ and CD8+ T cells that returned to near baseline levels prior to the next dosing cycle were observed to subsequently increase over the remaining dosing cycles (Figure S3). The increase in frequency of CD8+ T cells was much greater (from 12.7 to 26.7% by cycle 10) than that of the CD4+ T cells (from 3.8 to 7.7% by cycle 10). These increases in CD4+ and CD8+ T-cell frequencies were observed against a background of decreases in absolute lymphocyte count and CLL cells (Figure S2). The patient also showed a 69% decrease in the frequency of T_regs_ from baseline levels by the 10^th^ dosing cycle (Figure S3).

The patient continued to show improvement with a further reduction in tumor size of the left axillary lymph node noted on day 91 (cycle 3, day 14): 1.68 cm^2^, for a reduction of 70.93% from baseline (Figure S4). The patient’s platelet count had normalized (150 x 10^9^/L), white blood cell count was 26.06 x 10^9^/L and hemoglobin and other routine laboratory values were within normal range.

Additional dosing cycles produced further decreases in absolute lymphocyte count and white blood cell count (Figure S2); decreases in lymphadenopathy were evident sooner than changes in these peripheral cells. During the 5^th^ dosing cycle on day 158 (cycle 5 day 25) there was a 54.3% decrease of total tumor burden, but only a transient decrease (29.7%) in absolute lymphocyte count (7.1 x 10^9^/L on day 133, cycle 5 day 0). Reductions were sustained and a partial response was achieved one month later.

By the tenth dosing cycle on day 273 (cycle 10 day 0), a reduction from baseline in CLL cells (62.6%) and absolute lymphocyte count (43.9%) was noted and both exhibited a parallel decreasing trend. CT scan measurement taken on this day showed that bulky lymphadenopathy was decreased by 57.3%. Concordant decreases in absolute lymphocyte count and peripheral CLL cells were noted until cycle 12, after which time peripheral CLL cells showed a slight increase and absolute lymphocyte count trended slightly lower.

The patient continued to improve until the end of the trial. At the last study visit on day 392 (cycle 13 day 35), clinical assessment showed a maintained PR and the left axillary lymph node tumor showed further improvement measuring 0.75 cm^2^, for a reduction of 87.02% from baseline as confirmed by CT scan (5.78 cm^2^ vs. 0.75 cm^2^, baseline vs. week 56, respectively) (Figure S4). At this time, the patient had a 70% reduction in absolute lymphocytes and a 65.5% reduction in peripheral CLL cells. PR was maintained through six years following the final samalizumab dose, and the patient remains well (D.M., personal communication).

**Supplemental Table and Figure Legends**

| **Adverse Event**  **System Organ Class** | **Samalizumab Treatment Group** | | | |  |
| --- | --- | --- | --- | --- | --- |
| **Preferred Term** | **50 mg/m^2^ N = 4** | **100 mg/m^2^ N = 5** | **300 mg/m^2^ N = 3** | **400 mg/m^2^ N=3** | **Overall N = 26** |
|  |  |  |  |  |  |
| Blood and Lymphatic System Disorders | - | - | 2 | - | 3 (12%) |
| Anemia | - | - | 1 | 1 | 2 (8%) |
| Neutropenia | - | - | 1 | 1 | 2 (8%) |
| Thrombocytopenia | - | - | - | 1 | 1 (4%) |
| Eye Disorders | 1 | - | - | - | 1 (4%) |
| Visual Acuity Reduced | 1 | - | - | - | 1 (4%) |
| Musculoskeletal and Connective Tissue Disorders | 1 | - | - | - | 1 (4%) |
| Muscular Weakness | 1 | - | - | - | 1 (4%) |
| Infections and Infestations | - | 1 | - | - | 1 (4%) |
| Respiratory Syncytial Virus Infection | - | 1 | - | - | 1 (4%) |
| Skin and Subcutaneous Tissue Disorders | 1 | - | - | - | 1 (4%) |
| Rash | 1 | - | - | - | 1 (4%) |

**Table S1. Summary of Drug-Related Treatment-Emergent Adverse Events (Grade 3-4) reported by patients.**

**Figure S1. Serum concentration-time profiles of samalizumab.** Graphs show the mean serum concentration of samalizumab after the first intravenous administration at the indicated doses. Error bars represent the standard deviation.

Figure S2. Changes in CLL cells, absolute lymphocyte count, and white blood cells during 13 cycles of dosing in Patient 102-502. Time course of change in leukocyte subsets during multiple dosing with 400 mg/m^2^ samalizumab.

Figure S3. Changes in CD4+, CD8+, and Tregs during 13 cycles of dosing in Patient 102-502. The time course of change in the indicated T-cell subset populations during multiple dosing with 400 mg/m^2^ samalizumab.

**Figure S4. CT scans of a single lesion in Patient 102-502 before and after one dose of samalizumab demonstrate anti-tumor activity of samalizumab.** Serial scans of a single lesion in the left axillary lymph node (A) at screening on day -12, and (B) at the first post-dose assessment on day 28. Sentinel lesion is indicated by the red arrow and bi-dimensional measurements are shown in purple.

**Figure S1**

**Figure S2**

**Figure S3**

**Figure S4:**

**Day -12 (pre-dose) Day 28 (post-dose)**


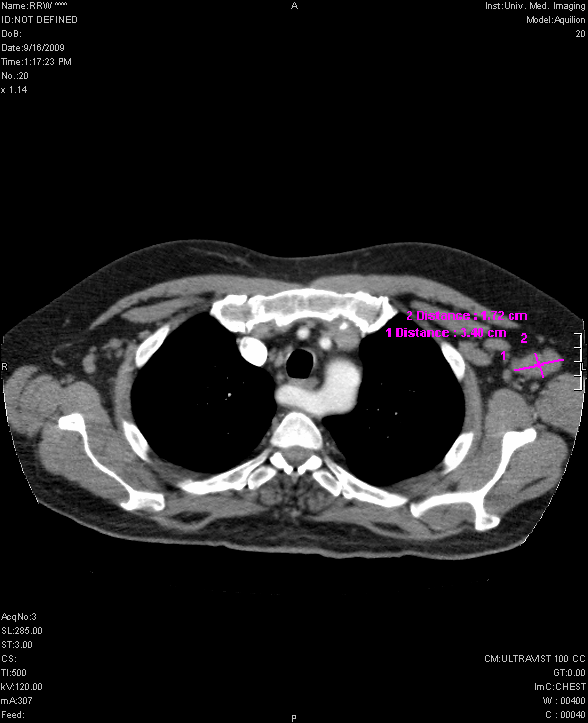

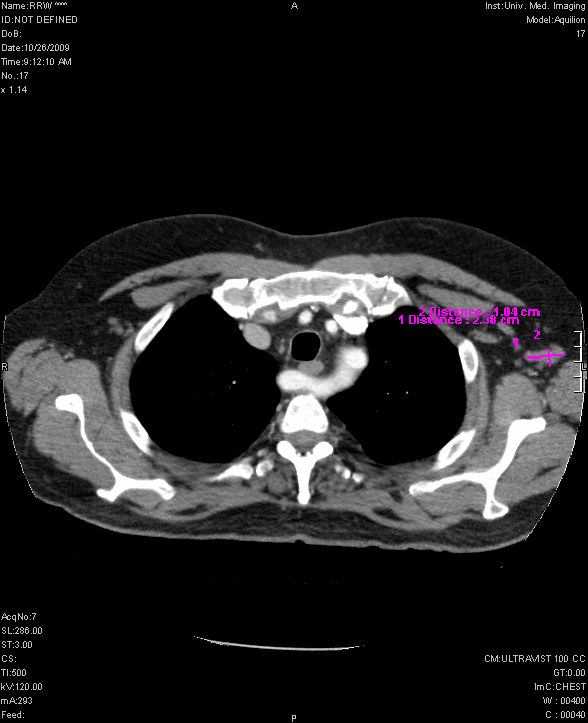

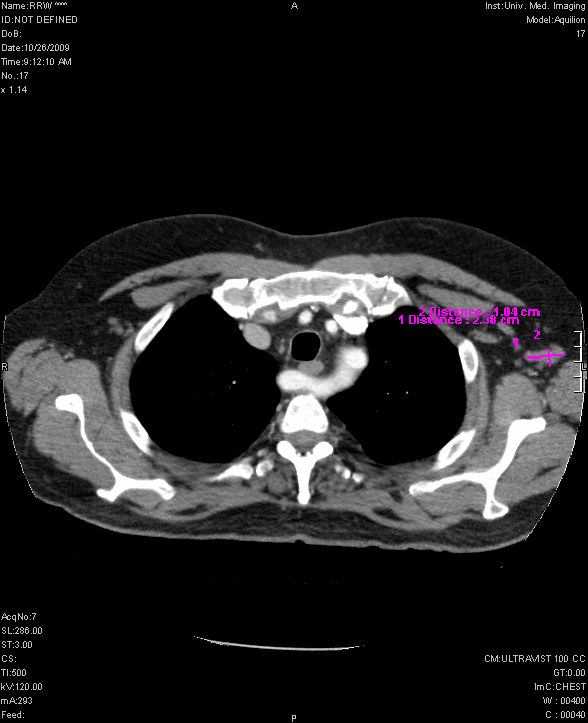

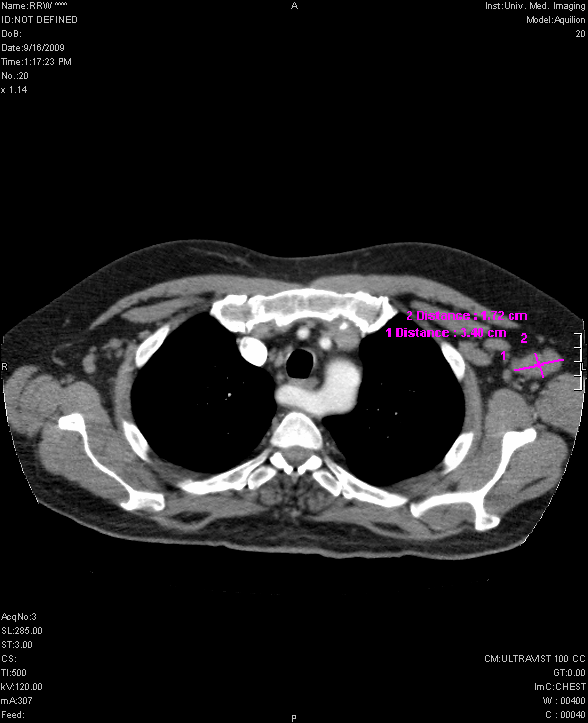

Supplement: Supplementary file 1 — Supplemental data on the Phase I Samalizumab trial of CLL and MM. (DOCX 799 kb) [file 40425_2019_710_MOESM1_ESM.docx]
